# Ordering of Zn-centered porphyrin and phthalocyanine on TiO_2_(011): STM studies

**DOI:** 10.3762/bjnano.8.11

**Published:** 2017-01-11

**Authors:** Piotr Olszowski, Lukasz Zajac, Szymon Godlewski, Bartosz Such, Rémy Pawlak, Antoine Hinaut, Res Jöhr, Thilo Glatzel, Ernst Meyer, Marek Szymonski

**Affiliations:** 1 Research Centre for Nanometer-Scale Science and Advanced Materials, NANOSAM, Faculty of Physics, Astronomy, and Applied Computer Science, Jagiellonian University, Lojasiewicza 11, 30-348 Krakow, Poland,; 2 University of Basel, Department of Physics, Klingelbergstrasse 82, 4056 Basel, Switzerland

**Keywords:** dye-sensitized solar cells, molecular nanostructures, phthalocyanines, porphyrins, rutile surfaces, STM imaging

## Abstract

Zn(II)phthalocyanine molecules (ZnPc) were thermally deposited on a rutile TiO_2_(011) surface and on Zn(II)*meso*-tetraphenylporphyrin (ZnTPP) wetting layers at room temperature and after elevated temperature thermal processing. The molecular homo- and heterostructures were characterized by high-resolution scanning tunneling microscopy (STM) at room temperature and their geometrical arrangement and degree of ordering are compared with the previously studied copper phthalocyanine (CuPc) and ZnTPP heterostructures. It was found that the central metal atom may play some role in ordering and growth of phthalocyanine/ZnTPP heterostructures, causing differences in stability of upright standing ZnPc versus CuPc molecular chains at given thermal annealing conditions.

## Introduction

There is an increasing interest in optoelectronic applications of organic molecular heterostructures which utilize inorganic substrates, such as titanium oxides. Recently various devices, such as light-emitting diodes [1], organic field effect transistors [2], and dye-sensitized solar cells [3], have been developed and commercialized. It is apparent that in almost all areas of utilization, the electronic properties of complex structures play a crucial role. In general, they depend not only on the characteristics of the individual building blocks, but are also sensitive to the organic–inorganic interface and the molecule orientation [4–5]. In this context, metal containing phthalocyanines and porphyrins are very often used for microscopic studies with the aim to understand molecule–molecule and molecule–substrate interactions [6–8]. The strength and physicochemical character of such interactions determine the system geometrical structure and electronic properties; hence, detailed knowledge is necessary for optimization of the optoelectronic device functionality. It was reported that a mixture of porphyrins and phthalocyanine has a profound impact on the photovoltaic parameters of dye-sensitized solar cells [9–10]. Therefore, the microscopic investigation on such systems is crucial.

One of the most popular nonmetal substrates used for investigation of molecular adsorption is titanium dioxide [11–12]. The most stable and the most studied face of TiO_2_ is the rutile (110) surface. In the context of adsorption studies, it is important to note that the (110) face of rutile usually contains numerous oxygen vacancies, often filled with hydroxy groups [13]. Those common surface defects are known to have an important effect on the molecule migration and surface diffusion barriers for Pd atoms [14–15], and as demonstrated by Kolmer et al., on the TiO_2_(011)-(2×1) surface, they play a very significant role in on-surface synthesis of polymers [16–17]. In the present work we use a TiO_2_(011)-(2×1) surface, since from our previous reports [5,12,18–22] we know that this rutile face offers higher mobility for molecular building blocks than the (110) one. This is most likely due to the fact that Ti atoms at this surface are 5-fold coordinated and relatively well screened from the surface molecules by protruding surface oxygen atoms [23–24]. We expect, therefore, better conditions for intermolecular ordering and less influence of the molecule–substrate interactions on the molecule assembly [5].

In our previous reports [5,18–19], we have shown that copper phthalocyanine (CuPc) molecules deposited on rutile (011) could form ordered planar nanostructures up to a complete monolayer coverage. At higher deposition, this wetting layer of planar molecules became covered with 2-dimensional (2D) molecular islands which could be stabilized and further ordered by thermal annealing at 150–200 °C. Although the structure of the thermally annealed CuPc islands could be characterized with low temperature (LT) scanning tunneling microscopy (STM), indicating that the molecules are predominantly upright-oriented, at room temperature, the molecular structures were unstable against the STM tip, precluding any high-resolution imaging. It was found, however, that this situation could be greatly improved if the wetting layer of the native CuPc molecules was substituted by Zn(II)*meso*-tetraphenylporphyrins (ZnTPP) [5]. The present work extends this study to Zn(II) phthalocyanines, not only to demonstrate the observations yet for the other system, but with the aim to find out whether the central metal atom plays any significant role in surface adsorption and ordering. Some indications that this could take place have been recently reported for B-passivated Si(111) substrates [25].

## Results and Discussion

### ZnPc adsorption and ordering

**Figure 1:**
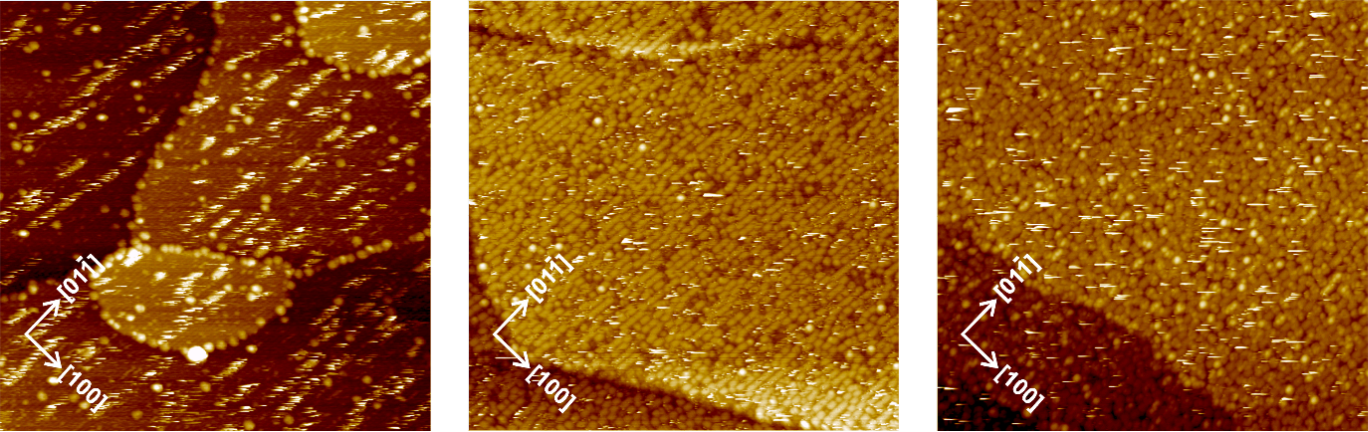
Adsorption of ZnPc molecules on the TiO_2_(011)-(2×1) surface. From left to right: empty state STM images of 0.1 ML, 0.9 ML, and 1.1 ML of ZnPc molecules respectively deposited on TiO_2_(011) surface at room temperature (*I* = 3 pA, *U* = 2 V). Image size: 100 × 100 nm^2^.

Figure 1 presents the constant current STM images of ZnPc molecules deposited at room temperature on the (2×1) reconstructed (011) face of rutile. At low coverage (≤1 ML, Figure 1, left panel), it is clear that the apparent geometrical arrangement of the molecules is governed by the alignment of protruding oxygen reconstruction rows (i.e., a [01−1] crystallographic direction. The contrast is rather unclear, already indicating relatively weak binding of the molecules to the substrate. Such apparent linear chains parallel to the [01−1] direction have been found and explained in our previous work on CuPc molecules [18]. At low coverage the molecules are perturbed by the scanning STM tip, causing a repetitive movement between the same surface barrier points during subsequent scans, such as surface hydroxy groups, terrace step edges, and/or stable molecules trapped on the surface. As a result, several apparent linear chains of ZnPc molecules along [01−1] are seen in addition to individual molecules decorating the terrace step edges.

At a coverage approaching 1 ML (see Figure 1, central panel), a stable structure of flat-laying molecules is formed preferentially oriented along the [01−1] direction, with some defects seen such as empty spots of missing molecules or misaligned molecules between the substrate reconstruction rows. The ordering of the ZnPc monolayer is similar to the CuPc one reported by Godlewski et al. [19] and Zajac et al. [5] but it seems to be more stable against the STM imaging at room temperature. This indicates that the molecule–substrate interaction related to the Zn central atom is stronger than for Cu-centered phthalocyanine. However, it is not strong enough to hamper the surface diffusion necessary for the observed level of surface ordering. Similar ordering is achieved also for coverages slightly exceeding the monolayer (Figure 1, right panel), with additional bright spots attributed to molecules present on top of the wetting layer.

**Figure 2:**
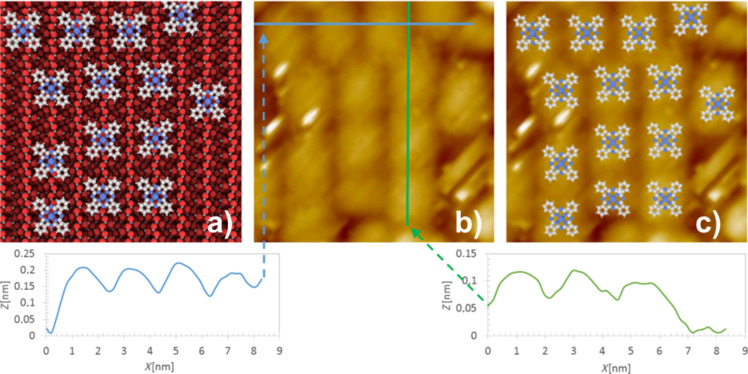
Geometrical characterization of 0.9 ML of ZnPc molecules on the TiO_2_(011)-(2×1) surface. (a) Illustration of the model of ZnPc molecule locations on the substrate closely reproducing the molecular contrast of the STM images; (b) and (c): high resolution empty state STM images (9×9 nm^2^) taken at RT. The linear profiles taken along [01−1] and [100] directions are shown below the STM images. The overlay of molecule locations with the STM image is shown in (c). STM scanning parameters are: *I* = 3 pA, *U* = 2.3 V.

High-resolution STM imaging of the ZnPc overlayer on the rutile (011) face clearly indicates molecular resolution achieved at room temperature (see Figure 2) and the linear cross-cuts along [01−1] and [100] directions allow for a precise geometrical characterization of the molecule configuration within the first surface overlayer. Similarly to previously reported observations for the CuPc wetting layer [18–19], the most likely locations of the ZnPc adsorption sites are over the oxygen protruding atoms of the substrate reconstruction rows. It is seen that the molecules are located over every second surface reconstruction zig-zag row accommodating well to the spacing available along the [100] direction (2 × 0.92 nm) (see the left profile in Figure 2b). The intermolecular distance along the rows is typically close to double of the nominal size of the free ZnPc molecule (2 × 1.19 nm), although larger intermolecular distances are observed too (see left chain of the molecules on the STM image in Figure 2). It is also seen that apart from the straight molecular lines, also chessboard-like structures are observed. A very similar level of ordering and stability at room temperature (RT) as observed for the ZnPc wetting layer has been recently reported by Olszowski et al. for ZnTPP [26].

**Figure 3:**
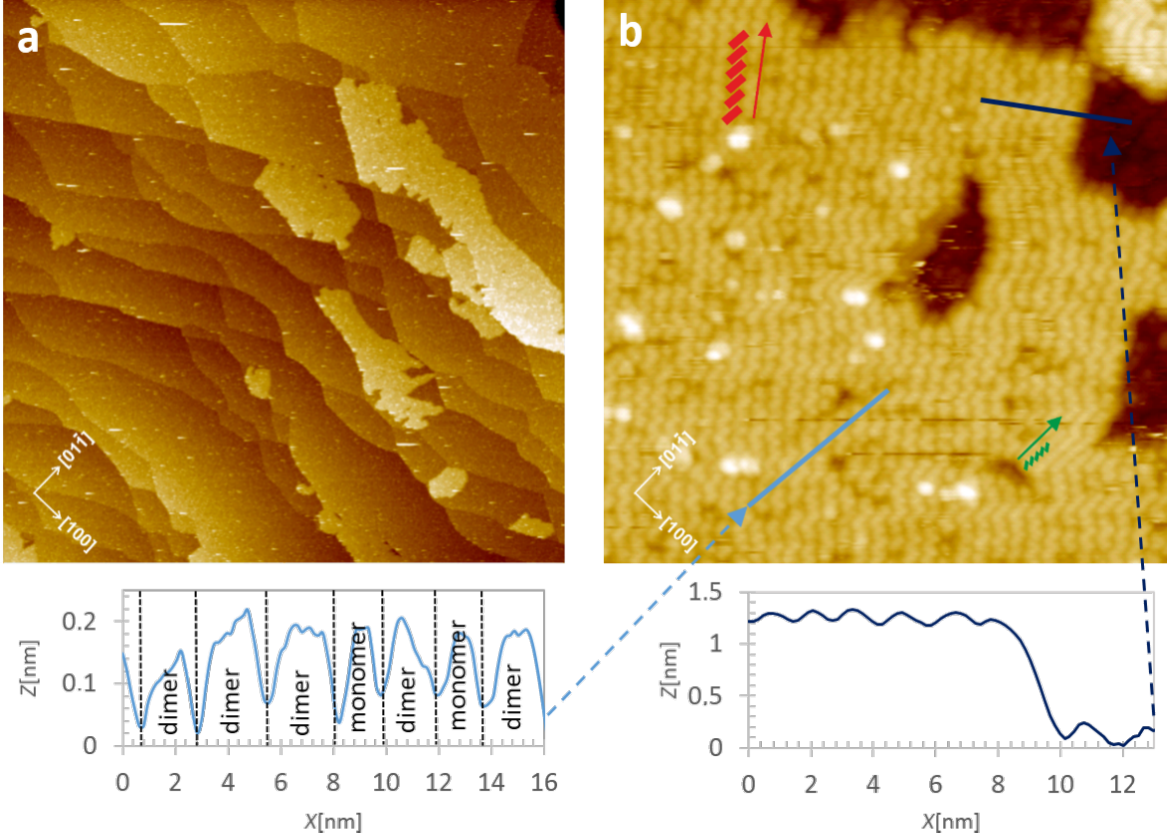
Geometrical characterization of 1.3 ML of ZnPc molecules on a TiO_2_(011)-(2×1) surface after deposition at 150 °C. (a) The overview 250 × 250 nm^2^ empty state STM image measured at RT. (b) The high resolution empty state STM images (50 × 50 nm^2^) taken at room temperature. Red and green arrows and rectangles indicate the ordering directions and the basic molecular building blocks, i.e., upright standing molecules. The linear profiles taken along the direction of the paired molecular building blocks (“bricks”) and across the island edge are shown below the STM image. In two cases the building blocks are smaller and most likely consist of monomers only. The STM scanning parameters are: *I* = 3 pA, *U* = 2 V.

Increasing the ZnPc molecule coverage above 1 ML results in the appearance of rather unstable molecules in the second layer, which form a smeared pattern under STM imaging (see Figure 1, right panel). This indicates that at RT the molecules of the second layer are rather weakly bound to the wetting layer. The situation can be significantly changed by thermal annealing. The application of 30 min of postdeposition thermal treatment at 150 °C transforms the system into randomly distributed 2D islands which are sufficiently stable to be imaged by STM. A more uniform distribution of 2D molecular islands could be obtained by performing the deposition of the ZnPc molecules at elevated temperature as presented in Figure 3a,b. It is important to note that annealing the ZnPc overlayer structures at 200 °C or higher leads to island disintegration and thermal desorption. The linear profiles across the island edges (see Figure 3b) reveal that the islands consist of closely packed molecules in an upright configuration. The apparent height of the molecules is about 1.2 nm which corresponds well with the dimension of a free ZnPc molecule of 1.19 nm. Similarly, as observed in our recent work on CuPc adsorption [5], two possible arrangements of the molecules within the annealed islands are seen. The arrangement of the first phase, marked by green rectangles in Figure 3b, consists of closely packed, tilted monomolecular building units aligned along the [01−1] crystallographic direction (i.e., along the substrate reconstruction rows). As discussed above, that direction is also adopted by the flat laying molecules of the wetting layer on which the 2D islands are formed.

Likely due to the thermal activation, the molecules could reach a stable minimum energy configuration characterized by a balance between strong mutual interaction between the molecules in the upright configuration and a relatively weak coupling to the wetting layer. From the molecular resolution of the image in Figure 3b and the STM images of other ZnPc islands grown on the wetting layer, we know that the apparent width of the closely packed molecular chain is less than 1 nm, so each chain could fit the spacing between two adjacent reconstruction rows of the TiO_2_ substrate. From our previous work on the LT-STM imaging of CuPc islands grown under similar conditions, we found that the upright standing CuPc molecules are packed roughly every 1 nm along the closely packed molecular chains. Since the geometrical size of both molecules is almost the same, we assume that the ZnPc spacing along the chain is roughly 1 nm too. Therefore, in our tentative geometrical model of the closely packed phase of the ZnPc island growth (the phase II in the terminology of [5]), we simply postulate that the chains are aligned along the reconstruction rows and arbitrarily tilted to obtain the required spacing and geometrical width (less than 1 nm). This type of molecular arrangement has been also proposed for CuPc molecules grown on ZnO [27].

**Figure 4:**
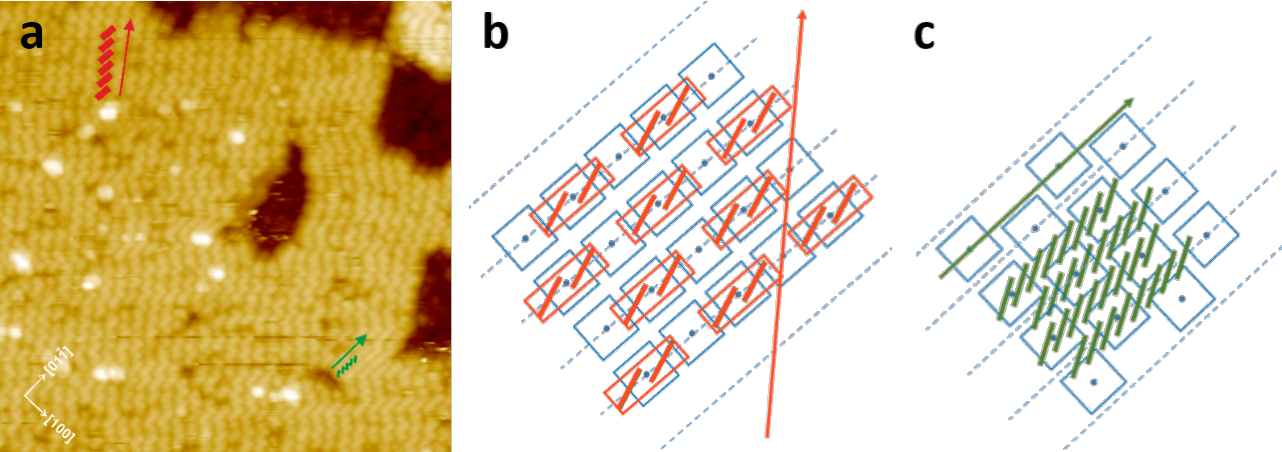
The tentative structural model of the first (b) and the second (c) phases of the molecular chain arrangement within ZnPc islands grown on the ZnPc wetting layer, demonstrating the molecular arrangement of the basic building blocks as identified in the STM image from panel (a). The blue squares with the central point denote flat laying molecules of the wetting layer, red rectangles containing 2 red lines denote the basic building blocks of the phase I structure, green rectangles represent the upright standing molecules of the phase II structure, dotted blue lines denote the direction of the every second protruding oxygen reconstruction rows on the (011)-(2×1) TiO_2_ substrate. Note that the flat laying molecules are distributed every second reconstruction row as described above. The STM scanning parameters for the image in (a) are: *I* = 3 pA; *U* = 2 V.

The dominant structure within the islands, however, appears as wider, slightly meandering rows of tilted brick-like units, each one large enough to contain a pair of ZnPc molecules in an upright position (the phase I in the terminology of [5]). The corresponding structure is marked in Figure 3b by red rectangles. It is seen that the extension of those paired molecular rows goes along the direction forming the fixed angle of 44 ± 2° with respect to the direction of the substrate reconstruction rows (red arrow in Figure 3b). The geometrical model of both phases is illustrated in Figure 4b,c. The molecular building blocks of the second layer are tentatively located on the wetting layer of flat laying ZnPc molecules (see blue squares in Figure 4) based on the apparent structure of the STM image from Figure 4a and the symmetry consideration for the building blocks with respect to the underlaying layer.

It appears that the most symmetric molecular arrangements for the phase I could be obtained assuming the rectangular structure of the wetting layer and for the phase II by assuming the chessboard structure of the flat laying ZnPc. Both possibilities for the wetting layer were found experimentally as described above. The tilt of the individual upright ZnPc molecular planes with the respect to the substrate reconstruction axis is somewhat arbitrarily adjusted to comply with the apparent widths of the molecular chains in both phases (about 1 nm). From the phase I model, it is also apparent that the alternate direction for the paired chain extension is possible, that is, at the angle symmetric with respect to the substrate reconstruction rows. Such orientation of the chains was observed experimentally on other ZnPc islands and both orientations are also seen within the same island in the case of the CuPc/ZnTPP heterostructure presented later in this work.

In summary, we suggest that the phase I versus phase II arrangements are moderated by the rectangular versus chessboard organization of the underlying wetting layer. Furthermore, this organization of the flat laying molecules on the substrate is quite likely dependent on the density and distribution of the hydroxy group defects on titania which could account for imperfection, defects, and phase to phase transitions within the same islands. Unfortunately, in the present experiment we were not able to control the initial distribution of the hydroxy groups on the titania surface. However, it was shown that the two equivalent N–H–O_br_ bonds are formed in the most favored configuration for the porphyrin molecules on TiO_2_(110) [28].

### Formation and ordering of ZnPc/ZnTPP heterostructures

**Figure 5:**
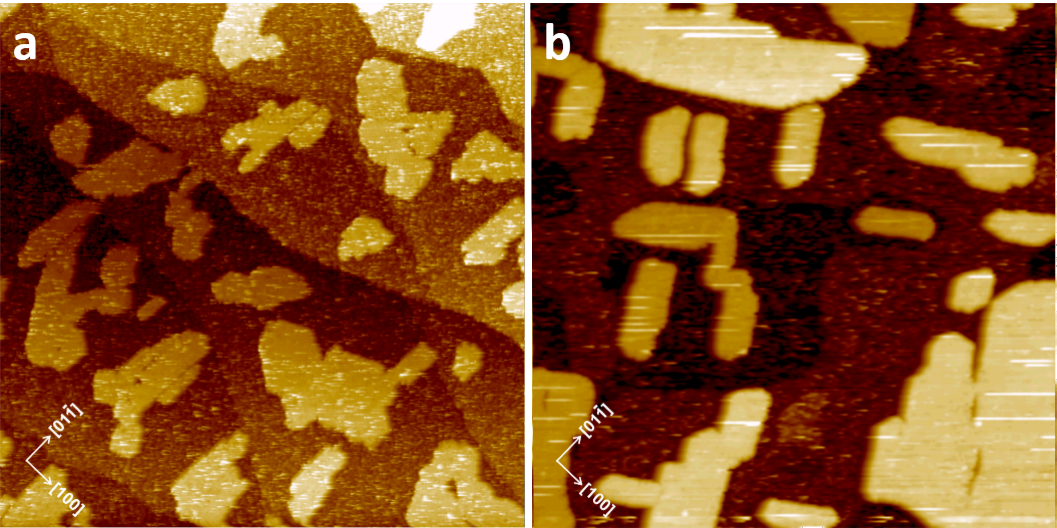
250 × 250 nm empty state STM images of ZnPc (a) and CuPc (b) structures formed on top of the ZnTPP wetting layer covering the TiO_2_(011)-(2×1) surface by thermal deposition of 0.1–0.2 ML of molecules and subsequent annealing at 150 °C. Scanning parameters: *I* = 5 pA; *U* = 2V.

In our recent work on CuPc molecule overlayers on rutile TiO_2_(011)-(2x1) [5] it was found that substituting the homomolecular wetting layer with the ZnTPP one resulted in greatly improved stability of such heterostructures, and high-resolution STM imaging was possible even at room temperature. In this work on ZnPc, we therefore apply the same ZnTPP wetting layer and compare the obtained results with the CuPc overlayer data. In all cases, the ZnTPP wetting layer is thermally annealed to 150 °C upon formation, in order to assure the same level of organization on the substrate prior to further deposition of molecules. In Figure 5 a,b the overview empty state STM images of ZnPc and CuPc structures are shown as formed on top of the ZnTPP wetting layer by thermal deposition of 0.1–0.2 ML of molecules and subsequent annealing at 150 °C.

**Figure 6:**
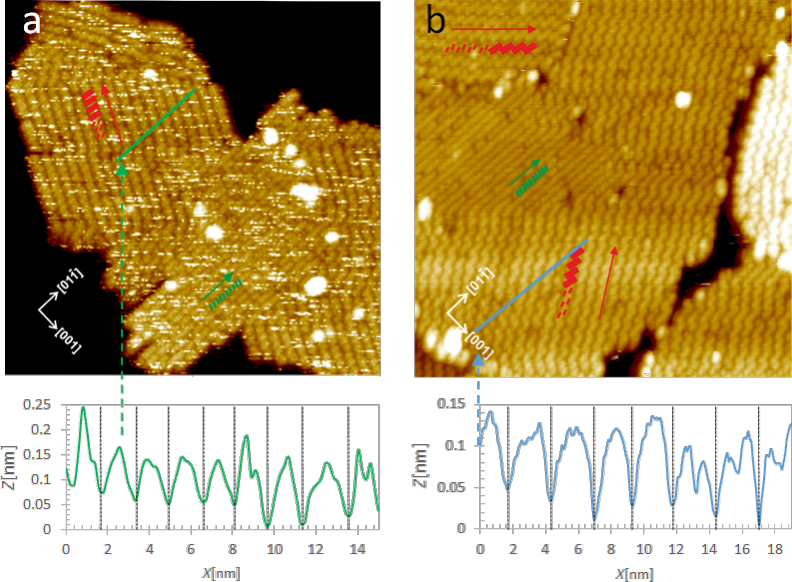
50 × 50 nm empty state STM images of ZnPc (a) and CuPc (b) structures formed on top of the ZnTPP wetting layer covering the TiO_2_(011)-(2×1) surface by thermal deposition of 0.1–0.2 ML of molecules and subsequent annealing at 150 °C. Arrows and rectangles indicate the ordering directions and the basic molecular building blocks, i.e., upright standing molecules. The profiles taken along those predominant directions of the molecular chains are shown below the STM images. Scanning parameters: *I* = 5 pA; *U* = 2V.

By comparing the shape of the overlayer islands we see an important difference: in the case of ZnPc (Figure 5a) more complex shapes of the islands are seen than for the predominantly rectangular shapes of the CuPc structures (Figure 5b). More detailed understanding of those differences could be realized from the high-resolution images and profiles presented in Figure 6. The two principal types of molecule arrangement (phase I of closely packed, tilted, individual molecule rows and phase II paired, molecular chains) are observed again, as in the case of homomolecular structures. However, for CuPc/ZnTPP there are two additional directions available for an extension of the paired molecular rows within the same island, that is, at angles of +44 ± 2° and −44 ± 2° with respect to the [01−1] direction of the substrate reconstruction rows. Furthermore, the close packed single molecule rows extending along the [01−1] are very seldom seen at the island edges, mostly cutting the sharp edges of the rectangles. Since the molecular rows essentially define the shape of the islands, in the case of CuPc, we predominantly observe almost regular rectangles, whereas ZnPc islands have edges aligned along paired molecule rows as well as single molecule chains, and often at more irregular directions perpendicular to the terminating rows/chains. Based on our STM images, it is not possible to determine whether the effect is caused by specific differences in the interaction of upright standing molecules (ZnPc versus CuPc) and the underlaying wetting layer, or just different activation energies for various forms of ordering at given thermal annealing conditions. Finally, we would like to note that our findings are not in any conflict with the recent report on the growth and ordering of metal phthalocyanine on B-passivated Si(111) surfaces [25]. Although it was indicated in that work that the central metal atom may play a significant role, the mechanism involved in such an interaction was concerned with the specific p–d orbital coupling between the localized Si substrate p_z_ states on the B-passivated Si(111) surface and the metal atom at the center of the flat laying phthalocyanine molecule. This type of mechanism is not applicable to the situation involving the upright standing ZnPc or CuPc molecules ordered on the flat laying ZnTPP molecules of the wetting layer.

## Conclusion

In this work we reported on the structures formed by Zn(II)phthalocyanines (ZnPc) on the bare and Zn(II)*meso*-tetraphenylporphyrin (ZnTPP) covered TiO_2_(011) surfaces. We demonstrated that initially ZnPc molecules form a quasi-ordered wetting layer on the titania substrate and that further deposition of molecules results in the formation of unordered clusters. However, those clusters could be transformed into ordered islands of upright ZnPc molecules by thermal annealing. Furthermore, we used ZnTPP molecules as wetting layers. Comparing the ordering of ZnPc islands on the ZnTPP buffer layer with the one previously observed for the CuPc/ZnTPP heterostructures [5], we found that although the basic building blocks of phthalocyanine second layer structures are essentially the same, the overall shape of the ZnPc islands is more complex than in the case of CuPc. This finding indicates that the central metal atom may play some role in ordering and growth of phthalocyanine/ZnTPP heterostructures. However, this seems to be rather due to differences in stability of upright standing ZnPc versus CuPc molecular chains at given thermal annealing conditions than to specific central metal atom interactions with the wetting layer molecules and/or titania substrate.

## Experimental

All experiments were performed in an ultra-high vacuum (UHV) system equipped with an Omicron variable temperature (VT) scanning tunneling microscope (STM), an ion gun, an effusion cell manufactured by Kentax GmbH, and a quartz crystal microbalance. The base pressure in the system was kept at 1 × 10^−10^ mbar.

**Figure 7:**
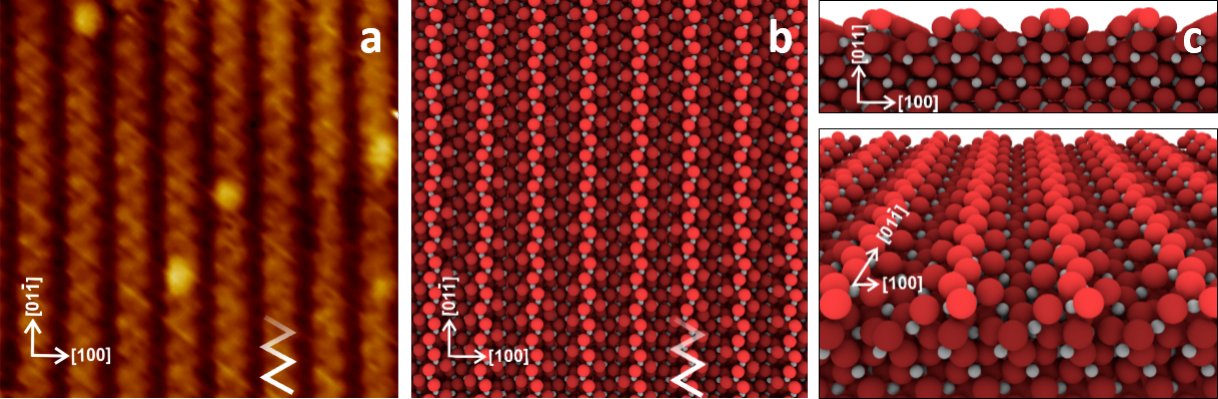
TiO_2_(011)-(2×1) reconstructed surface. (a) 9 × 9 nm empty state STM image; scanning parameters: *I* = 10 pA, *U* = 2 V. The white zig-zag pattern indicates the location of the protruding oxygen atoms forming the reconstruction row. The bright dots represent the surface hydroxy groups. (b) Top, (c) cross-section and perspective views of the surface layer “ball model” following the interpretation of the STM image proposed by Woolcot et al. [32]. Light red, red, and dark red circles indicate O; gray small dots indicate Ti atoms.

The rutile TiO_2_(011) samples purchased from MaTeck were prepared in a standard procedure by cycles of Ar^+^ ion sputtering at 1 keV energy, followed by annealing at 1040 K. The ball-model of the TiO_2_(011)-(2×1) surface reconstruction is presented in Figure 7 together with a high-resolution STM image of the same surface prepared in our UHV system as outlined above. The characteristic zig-zag rows of the protruding oxygen atoms extending along the [01−1] crystallographic direction are clearly visible both on the model drawing (light red balls) and at the high-resolution empty state STM image [29–32]. The additional bright corrugations seen on top of the reconstruction rows (seen in the STM image but not incorporated in the model) correspond to hydroxy groups [11,16].

**Figure 8:**
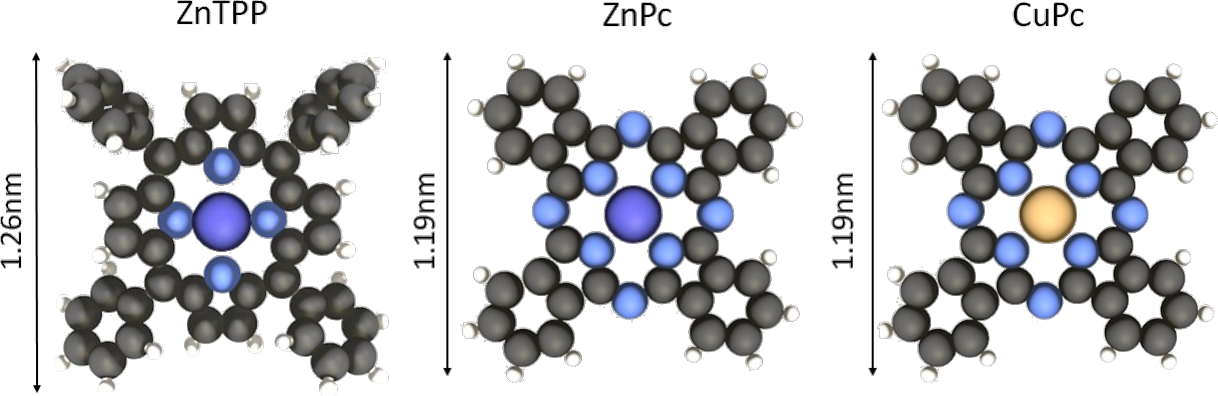
Chemical structure of the molecules used in the study (from left to right): Zn(II)*meso*-tetraphenylporphyrin (ZnTPP), Zn(II)phthalocyanine (ZnPc), and Cu(II)phthalocyanine (CuPc). Color coding: dark gray (carbon), light gray (hydrogen), blue (nitrogen), large violet (zinc), large yellow (copper).

The CuPc and ZnPc molecules were provided by Sigma-Aldrich, whereas ZnTPP by TriPorTech and was thermally evaporated from the Knudsen effusion cells with the rate calibrated at approximately 1 ML/h. The structural models of the molecules used in the present study are shown in Figure 8. Basically, we used a Zn atom containing porphyrin and phthalocyanine molecules with a 4-fold symmetry, but for comparison, the Cu-based phthalocyanines are discussed too.
